# Supplementary material for: Leukemia-intrinsic determinants of CAR-T response revealed by iterative in vivo genome-wide CRISPR screening
Source: Nat Commun. 2023 Dec 5;14:8048. doi: 10.1038/s41467-023-43790-2 (PMC10698189; doi:10.1038/s41467-023-43790-2)
Supplement: Supplementary file 1 — Supplementary Information [file 41467_2023_43790_MOESM1_ESM.pdf]

Leukemia-intrinsic determinants of CAR-T response revealed by iterative *in vivo* genome-wide CRISPR screening

Supplementary Figure 1

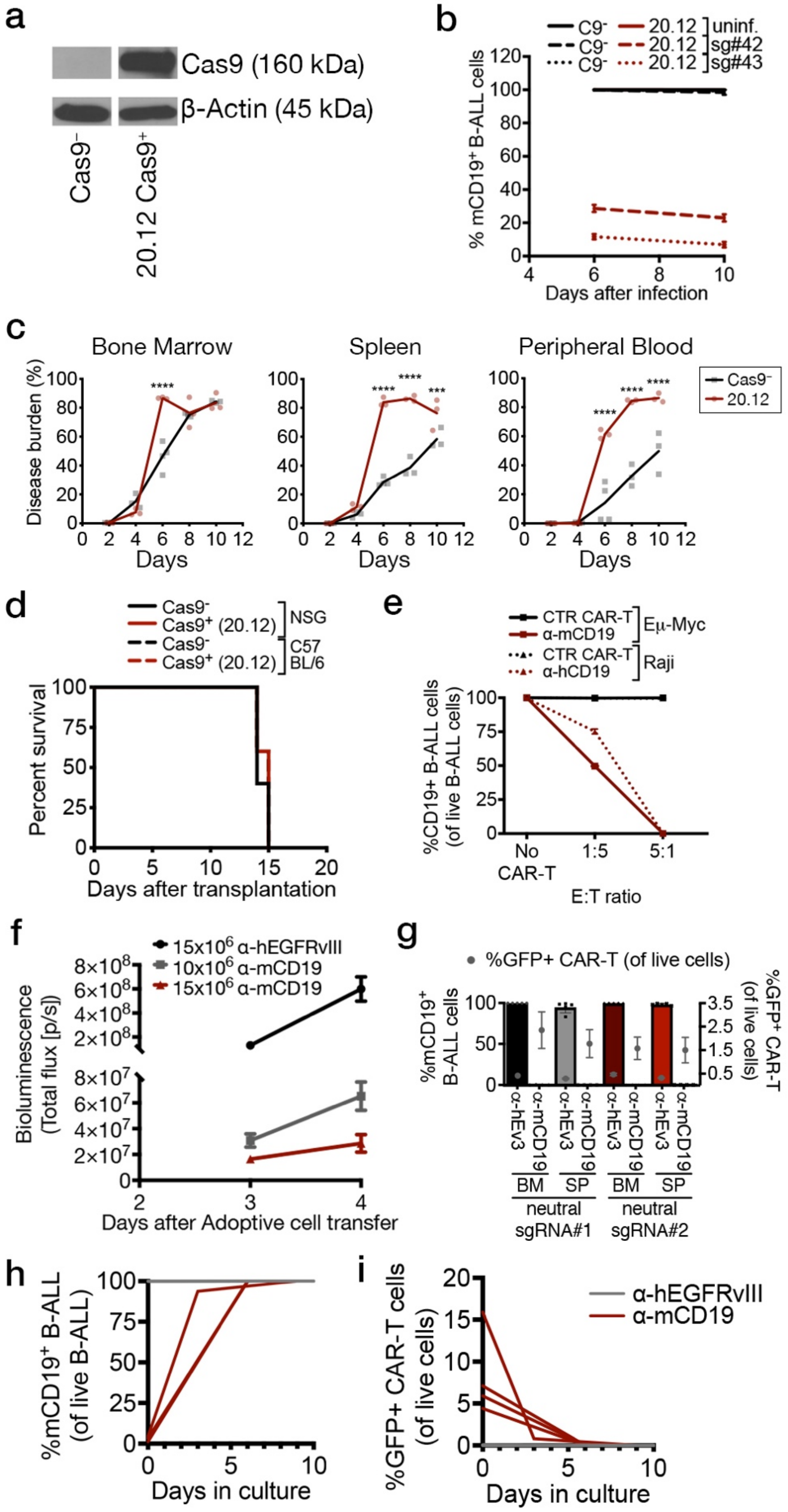

Supplementary Figure 1. Characterization of Cas9+ disease and target epitope loss after CAR-T treatment. (a) Cas9 expression in 20.12 cells assayed via western blot. (b) *In vitro* cut assay using two validated, non-overlapping guides against murine CD19 (mCD19) was used to determine Cas9 cutting efficiency in 20.12 cells. (c) Growth kinetics of $Tomato^+$ GFP^+ $Cas9^+$ 20.12 cells and non-Cas9 expressing parental cells in indicated hematopoietic organs of non-irradiated syngeneic B6 mice. Exact P values can be found in Extended Data 3. (d) Kaplan-Meier survival curves for non-irradiated immunocompetent (B6) and immunocompromised (NSG) mice transplanted with either $Cas9^-$ and $Cas9^+$ (20.12) disease. (e) Murine CAR-T cells targeting either mCD19 or human CD19 *in vitro* induce dose dependent target epitope loss in murine Eμ-Myc and human Raji cells, respectively. (f) Bioluminescence imaging of irradiated B6 mice treated with indicated CAR-T cell type and dose at indicated time after ACT. On the day of ACT (two days after injection of B-ALL disease), disease burden is undetectable by bioluminescence imaging. (g) B-ALL cells isolated from the organs of moribund mice that relapsed after treatment with 3.5 $x10^6$ CAR-T cells targeting mCD19 demonstrate ongoing CAR-T engraftment and complete target epitope loss. The proportion of mCD19 positivity in live B-ALL cells is shown with bar graphs and the left sided y-axis. The proportion of live cells that are GFP^+ CAR-T cells is shown with grey dots and the right sided y-axis. (h-i) Whole spleens of relapsed mice after treatment with $5x10^6$ indicated CAR-T cell type were dissociated, cultured *in vitro* and serially assessed for $mCD19^+$ B-ALL (h, showing proportion of live B-ALL cells that are mCD19 positive) and GFP^+ CAR-T cells (I, showing proportion of live cells that are GFP^+ CAR-T cells). As anti-mCD19 CAR-T cells are depleted from culture (i, red lines), B-ALL cells re-express mCD19 (h, red lines). All experiments were repeated at least once. Significance for survival experiments was determined using log-rank tests. For all other experiments, significance is determined using unpaired two-sided student's t-tests with Bonferroni correction for multiple comparisons. Data are mean±s.e.m.; n=4–8 mice per group. *P<0.05; **P<0.01; ***P<0.001; ****P<0.0001.

Supplementary Figure 2

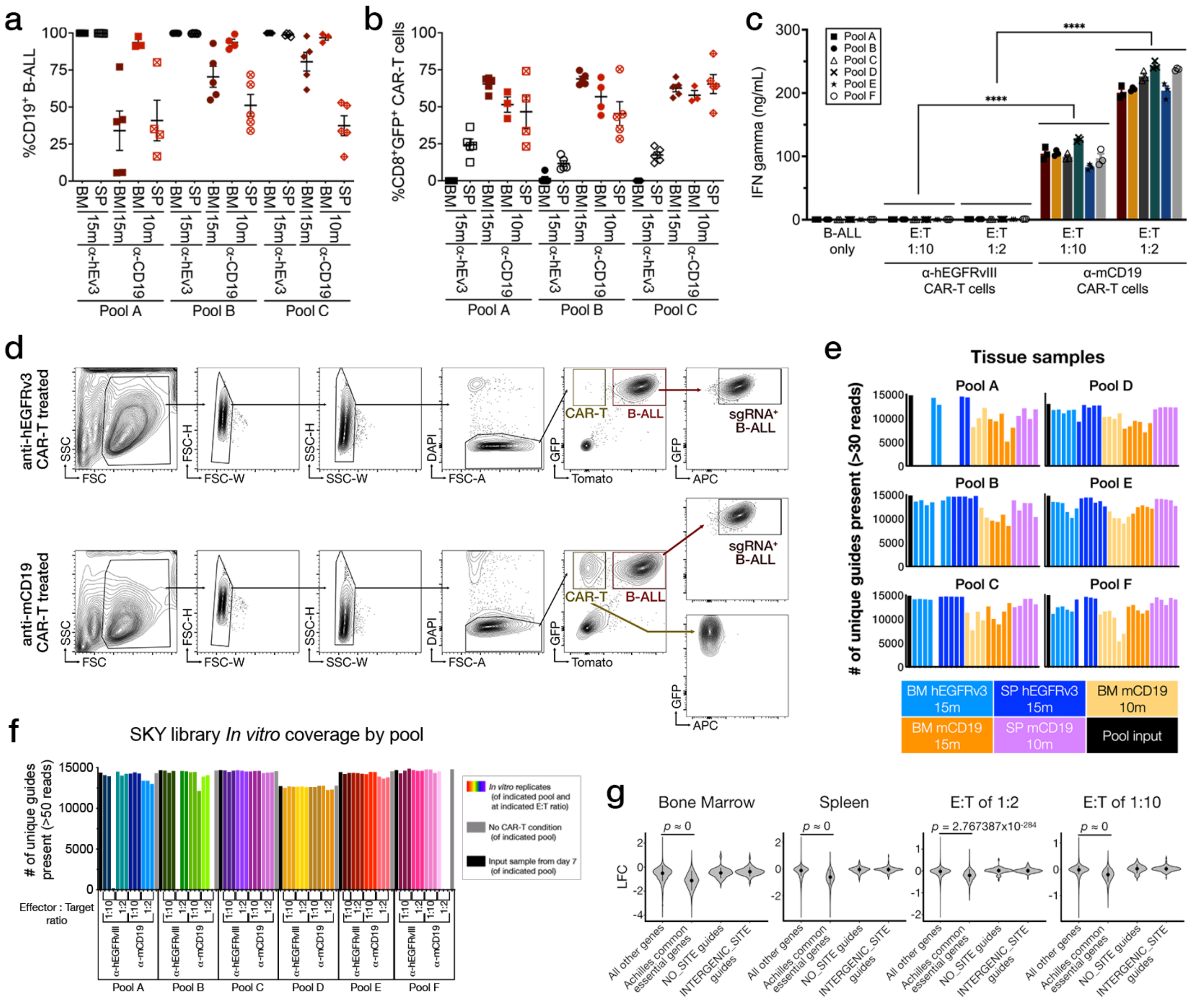

Supplementary Figure 2. *In vitro* and *in vivo* genome-wide CRISPR/Cas9 primary screens show comprehensive representation of sgRNA libraries and capture differences in sgRNA behavior between control and anti-mCD19 CAR-T treated mice. (a) Proportion of live B-ALL cells that are mCD19$^+$ in the spleen (SP) or bone marrow (BM) of relapsed mice after treatment with the indicated CAR-T cell and dose. (b) Proportion of GFP$^+$ persistent CAR-T cells (amongst all live CD8+ T-cells) in the spleen (SP) or bone marrow (BM) of relapsed mice after treatment with the indicated CAR-T cell and dose. (c) IFN**γ** release quantified via ELISA in cell culture supernatant collected 24 hours after *in vitro* kill assays with murine B-ALL cells. Anti-mCD19

CAR-T cells release significantly more IFN**γ** when exposed to their target antigen, as compared to control CAR-T cells. Differences between the two batches of CAR-T cells used in the primary screens (experiment 1: pools A-C; experiment 2: pools D-F). could not be detected using this assay. P values are <0.0001 for all comparisons shown. (d) FACS sorting scheme used to isolate live, guide bearing ($sgRNA^{+}$) B-ALL cells during both the primary and validation screens. (e) sgRNA library representation in each pooled library in the *in vitro* primary screen samples. Each bar shows the number of sgRNAs with more than 30 reads in the sequencing sample for a given pool. (f) Same as (e), but for sgRNA library representation in each pooled library in the *in vivo* primary screen samples. (g) Violin plots showing relative depletion/enrichment of control sgRNAs used in the primary screen. *P*-values are calculated from Wilcoxon rank-sum tests for comparison between pairs of group means. Note that in some cases exact *p*-values cannot be determined due to near-zero values and ties in data and are reported as approximately zero. For (a-c), data are mean±s.e.m., n=3–6 mice per group, as indicated by individual data points in (a-b) and significance is determined using unpaired two-sided student's t-tests with Bonferroni correction for multiple comparisons. In (e), each bar indicates a single mouse. In (f), each bar represents a single replicate. *P<0.05; **P<0.01; ***P<0.001; ****P<0.0001.

Supplementary Figure 3

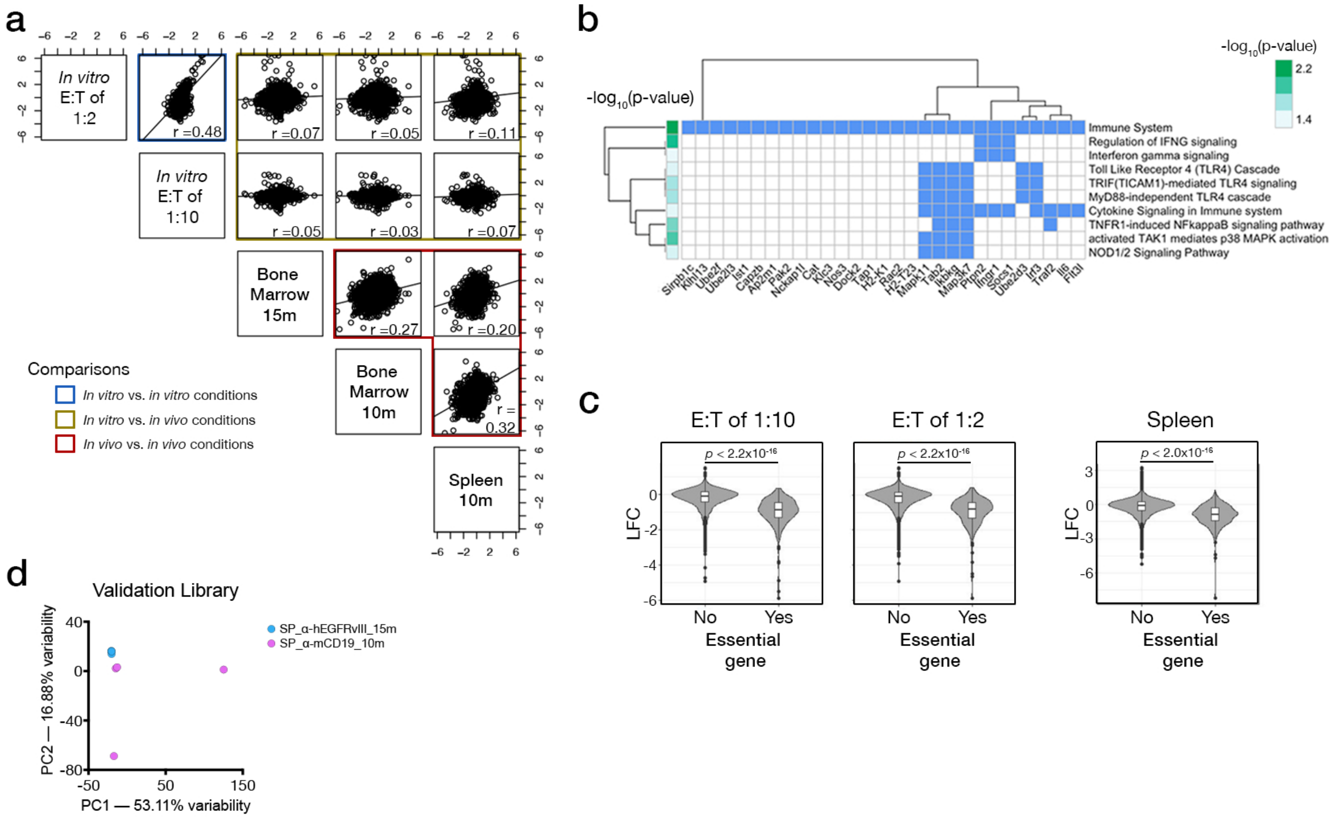

Supplementary Figure 3. Both genome-wide and validation CRISPR/Cas9-mediated screens demonstrate robust quality control characteristics. (a) Pair-wise correlations between each arm of the primary screens. Top gene hits from both *in vitro* screen conditions are correlated with one another (blue box). Top gene hits from all *in vivo* screen conditions show some correlation (red boxes). However, in all cases, top hits from *in vitro* versus *in vivo* conditions are poorly correlated to one another and demonstrate weak Pearson correlation coefficients closer to 0 (gold boxes). (b) Enrichment of KEGG terms within genes showing top sgRNA depletion in the *in vivo* arms of the screen. Shown is a heatmap of participation of the top genes in each enriched pathway. Significance was calculated with hypergeometric tests corrected for multiple hypothesis testing to determine whether pathway terms are overrepresented among *in vivo* hits. (c) Violin plots showing relative depletion/enrichment of control sgRNAs used in the validation screen. *P*-values are calculated from Wilcoxon rank-sum tests for comparison between pairs of group means. (d) *In vivo* validation screen samples projected onto the first 2 principal components of input-normalized sgRNA count matrix. The two experiments comprising the primary screen were completed one time each. The validation library was also completed

once. For the validation library, each *in vitro* screening condition was completed in triplicate and n = 4 mice per treatment group in the *in vivo* arm.

Supplementary Figure 4

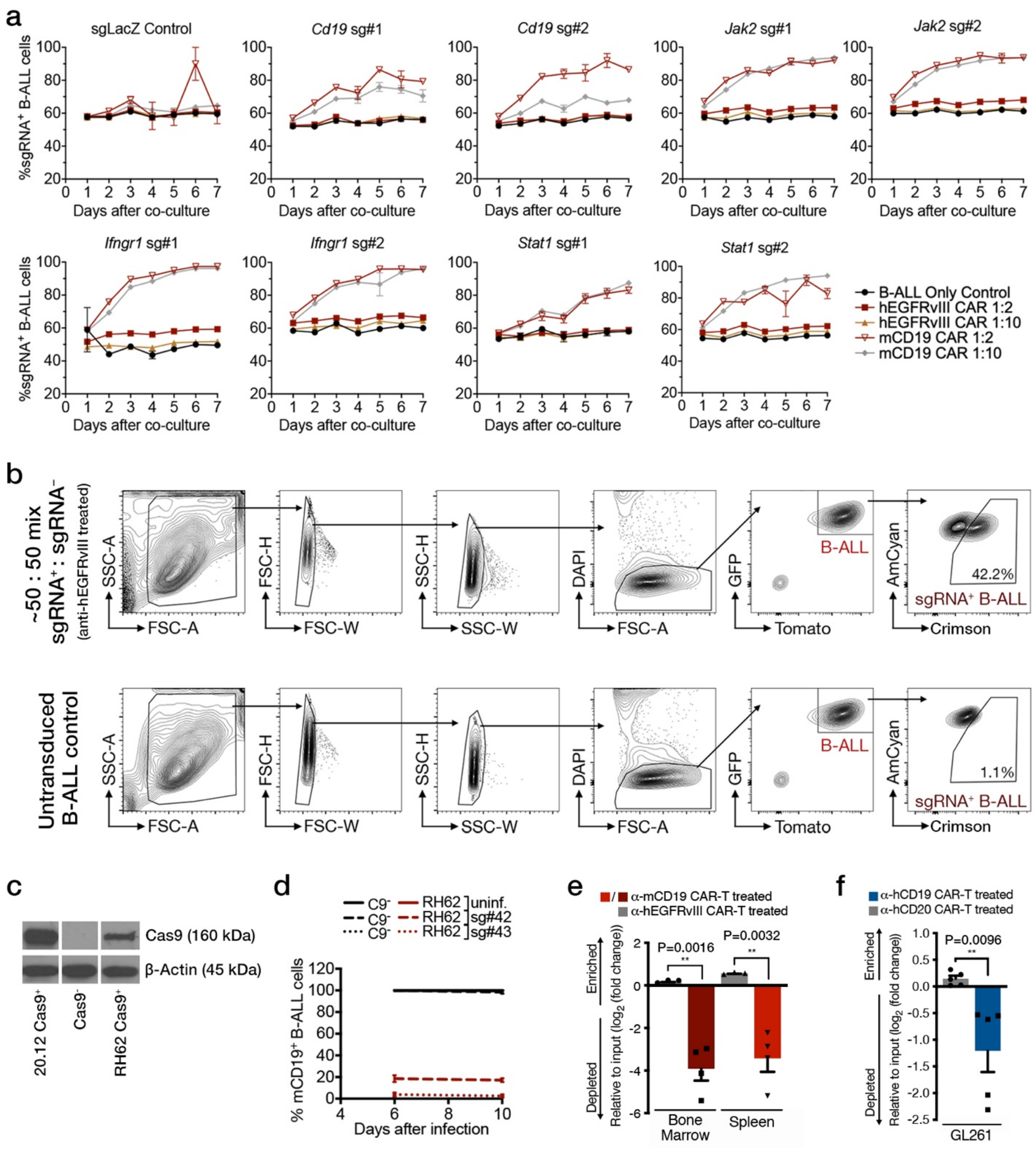

Supplementary Figure 4. *In vitro* cytotoxicity assays in cells deficient for components of the IFNγR/JAK/STAT pathway do not capture *in vivo* sensitization phenotypes to CAR-T therapy. (a) *In vitro* competitive assays completed in cells transduced with guides against the

indicated IFNγR/JAK/STAT pathway or control gene and treated with the indicated CAR-T cell type at two E:T ratios. Data shown is from flow cytometry analyses examining the proportion of live B-ALL cells that are APC^+ and therefore, also guide-bearing. (b) Examples of the flow cytometry analysis scheme used for these experiments, along with untransduced B-ALL control cells used to guide gate placement. (c) Western blot showing that Tomato+ RH62 B-ALL cells used for all validation experiments express Cas9 protein. (d) *In vitro* cut assay using two validated, non-overlapping guides against murine CD19 (mCD19) was used to determine Cas9 cutting efficiency in RH62 cells. Six days after the introduction of sgRNAs, more than 75% of all RH62 cells are $mCD19^-$, as assayed with flow cytometry. (e) *In vivo* competitive assays repeated in immunocompetent Cas9 transgenic mice again demonstrate specific log-fold depletion of $Cas9^+$ RH62 B-ALL cells lacking IFNGR1 after treatment with anti-mCD19 CAR-T cells (n=4 mice) in both the bone marrow (left) and the spleen (right), as compared to mice treated with control anti-hEGFRvIII CAR-T cells (n=3 mice). (f) *In vivo* competitive assays (n=5 mice per group) in a murine syngeneic orthotopic transplant model of glioblastoma multiforme (GBM, GL261 cells) demonstrate specific log-fold depletion of $Cas9^+$ GL261 cells lacking IFNGR1 after treatment with anti-mCD19 CAR-T cells. All *In vitro* experiments represented in this figure were repeated three times. Significance was determined using unpaired two-sided student's t-tests with Bonferroni correction for multiple comparisons. Data are mean±s.e.m. *P<0.05; **P<0.01; ***P<0.001; ****P<0.0001.

Supplementary Figure 5

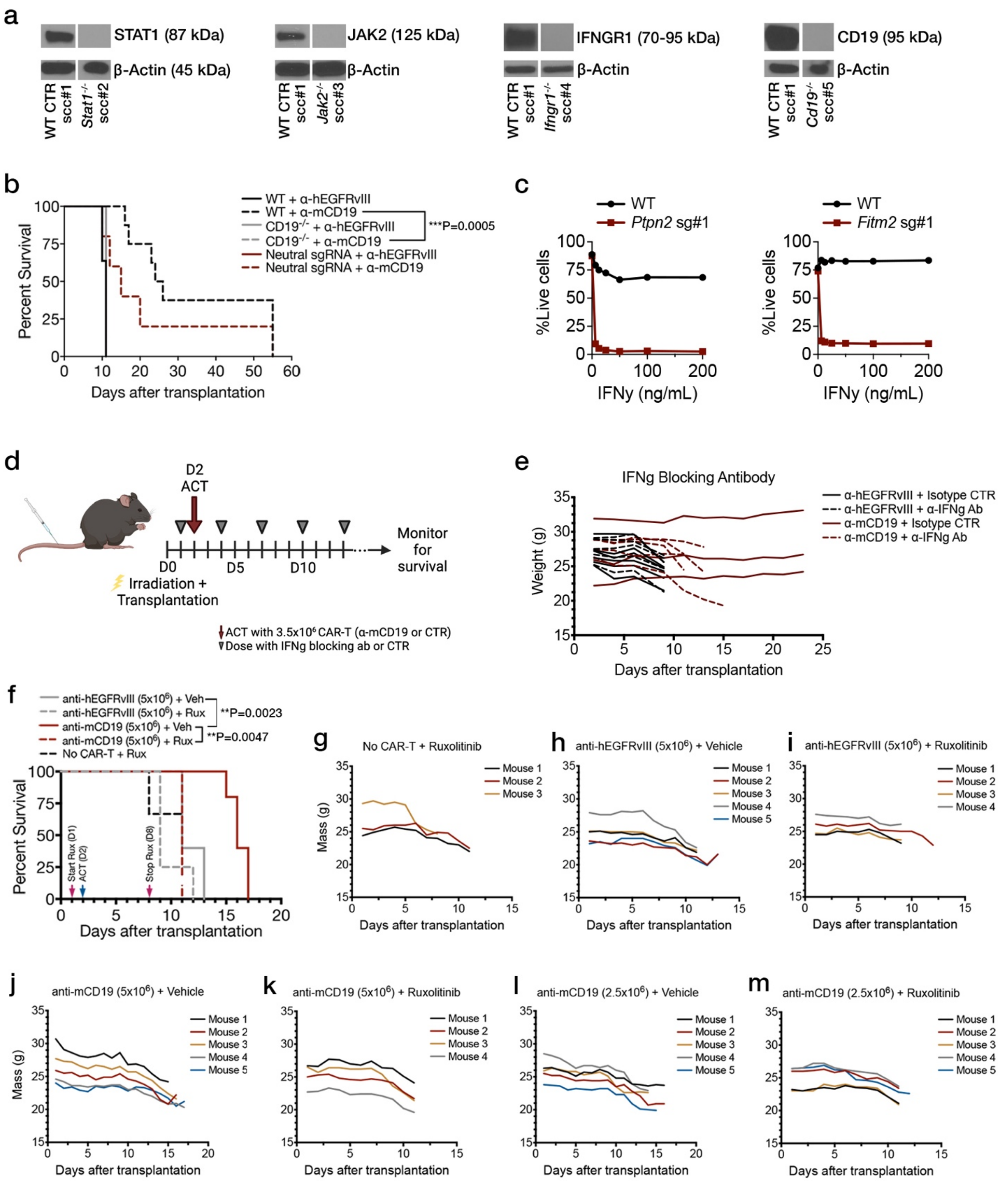

Supplementary Figure 5. Generation of single cell B-ALL clones deficient in members of the IFNγR/JAK/STAT pathway and animal weight monitoring for pharmacological experiments targeting IFNγR/JAK/STAT signaling. (a) Western blot analysis of single cell clones (scc) deficient in indicated gene and used in survival validation experiments. (b) Mice transplanted with parental (WT) or neutral control cells do not significantly differ in their survival after treatment with anti-mCD19 CAR-T cells, while mice transplanted with *Cd19*$^{-/-}$ B-ALL cells show no life extension. n=5-8 mice per group. (c) An *in vitro* survival assay in which WT, *Ptpn2*$^{-/-}$ or *Fitm2*$^{-/-}$ cells were treated with indicated doses of IFN**γ** for 48 hours and then assessed for viability. (d) Layout and treatment schedule for anti-Ifng blocking antibody experiment. (e) Mouse weight monitoring over time during co-treatment with CAR-T cells and anti-IFN**γ** blocking antibodies shows no additional toxicity during combination therapy. (f) A Kaplan-Meier curve showing overall survival in leukemia bearing mice treated with control or 5.0×10^6 anti-mCD19 CAR-T cells in the presence or absence of of Ruxolitinib, a JAK1/2 inhibitor. Exact n per group in indicated in (g-m). (g-m) Mouse weight monitoring over time during co-treatment with CAR-T cells and Ruxolitinib shows no additional toxicity during combination therapy. Data are exact mass in grams over time and the number of mice per group is indicated in each panel representing that group. Survival experiments were repeated three times. Pharmacologic experiments were completed once. Significance for survival experiments was determined using log-rank tests. *P<0.05; **P<0.01; ***P<0.001; ****P<0.0001.

Supplementary Figure 6

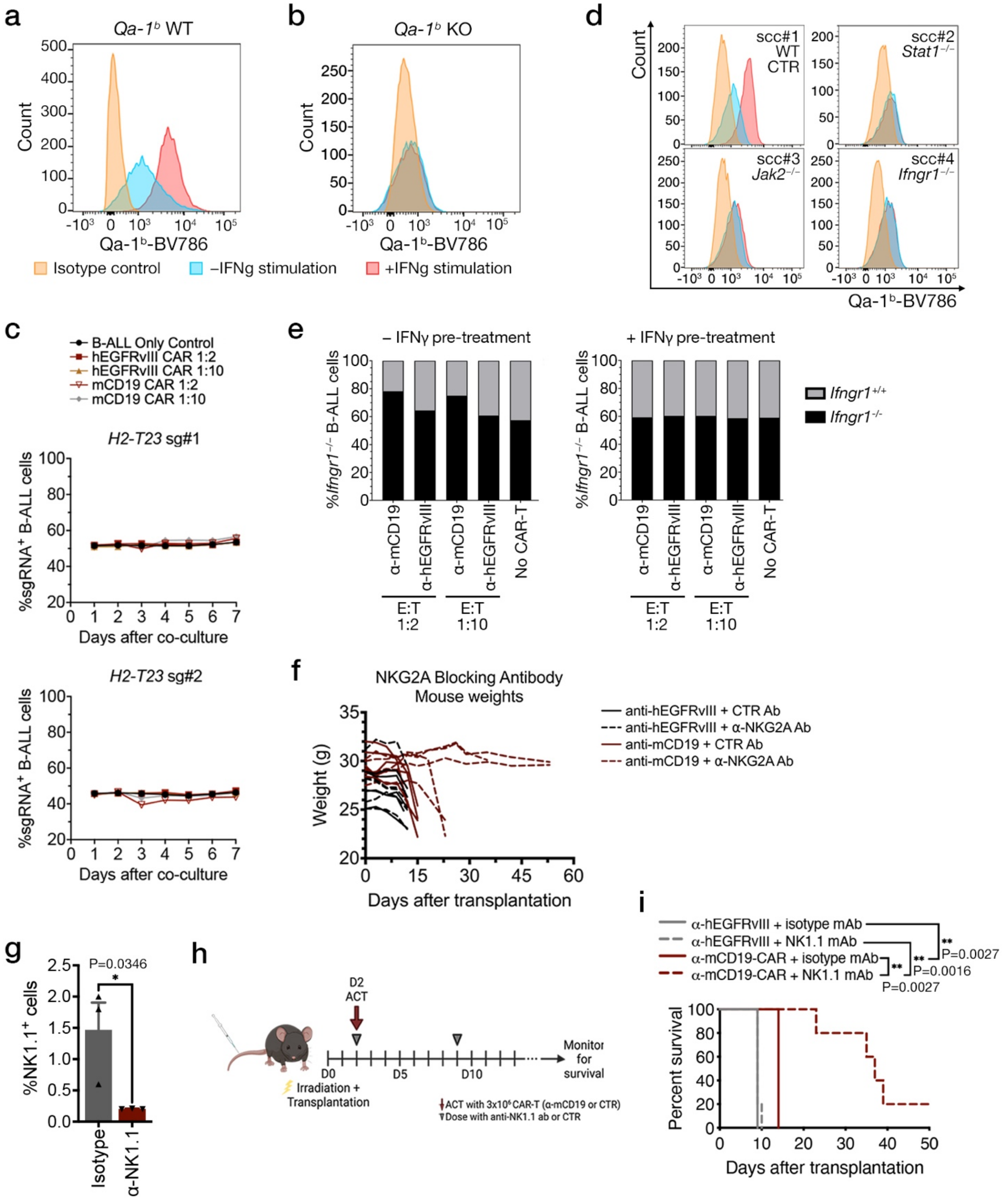

Supplementary Figure 6. Loss of Qa-1^b does not sensitize B-ALL cells to CAR-T therapy *in vitro*. (a-b) FACS histograms showing Qa-1^b expression on cells transduced with a control sgRNA (*Qa-1^b* WT) or *Qa-1^b*-deficient cells after stimulation with IFN**γ**. c) *In vitro* competitive assays completed in cells transduced with sgRNAs against *H2-T23* and treated with the indicated CAR-T cell type at two E:T ratios. Controls for this experiment can be found in Supplementary Figure 4a. Data are mean±s.e.m**.** (d) Expression of Qa-1^b-BV786 after *in vitro* stimulation with IFN**γ** of single cell clones (scc) deficient in indicated components of the IFN**γ**R/JAK/STAT pathway, as assayed using flow cytometry. Wildtype (WT) control single cell clones were also generated and assayed simultaneously. Representative data for a control WT clone is shown. Color key is identical to that in (a, b). (e) *In vitro* competitive assays in which *IFNG*$^{-/-}$ B-ALL cells were mixed at a 50:50 ratio and treated with anti-mCD19 CAR-T or anti-hEGVRvIII CAR-T cells at the indicated ratios. Cells were either pre-treated with exogenous recombinant IFN**γ** (right) or left untreated with exogenous recombinant IFN**γ** throughout the experiment (left). The graph shows the percentage of *IFNG*$^{-/-}$ cells (black) versus control *IFNG*$^{+/+}$ cells (gray) following treatment with the indicated CAR-T therapy type. (f) Mouse weight monitoring over time during co-treatment with CAR-T cells and NKG2A blocking antibody. Data are exact mass in grams over time and n=5 mice per group. (g) A graph showing the percentage of NK cells (as measured by NK1.1 positivity) in spleen 24 hours after treatment with an anti-mNK1.1 or isotype control antibody (n=3 mice per group). (h) Layout and treatment schedule for anti-NK1.1 NK cell depletion experiment. (i) Kaplan-Meier curves showing overall survival in mice treated with either anti-NK1.1 or isotype control antibodies in combination with either no CAR-T cells, anti-mCD19 CAR-T cells or control anti-hEGFRvIII CAR-T cells, as indicated (n=5 mice per group). Significance for survival experiments was determined using log-rank tests. For all other experiments, significance is determined using unpaired two-sided student's t-tests with Bonferroni correction for multiple comparisons, if applicable. Data are mean±s.e.m..*P<0.05; **P<0.01; ***P<0.001; ****P<0.0001.

Supplementary Figure 7

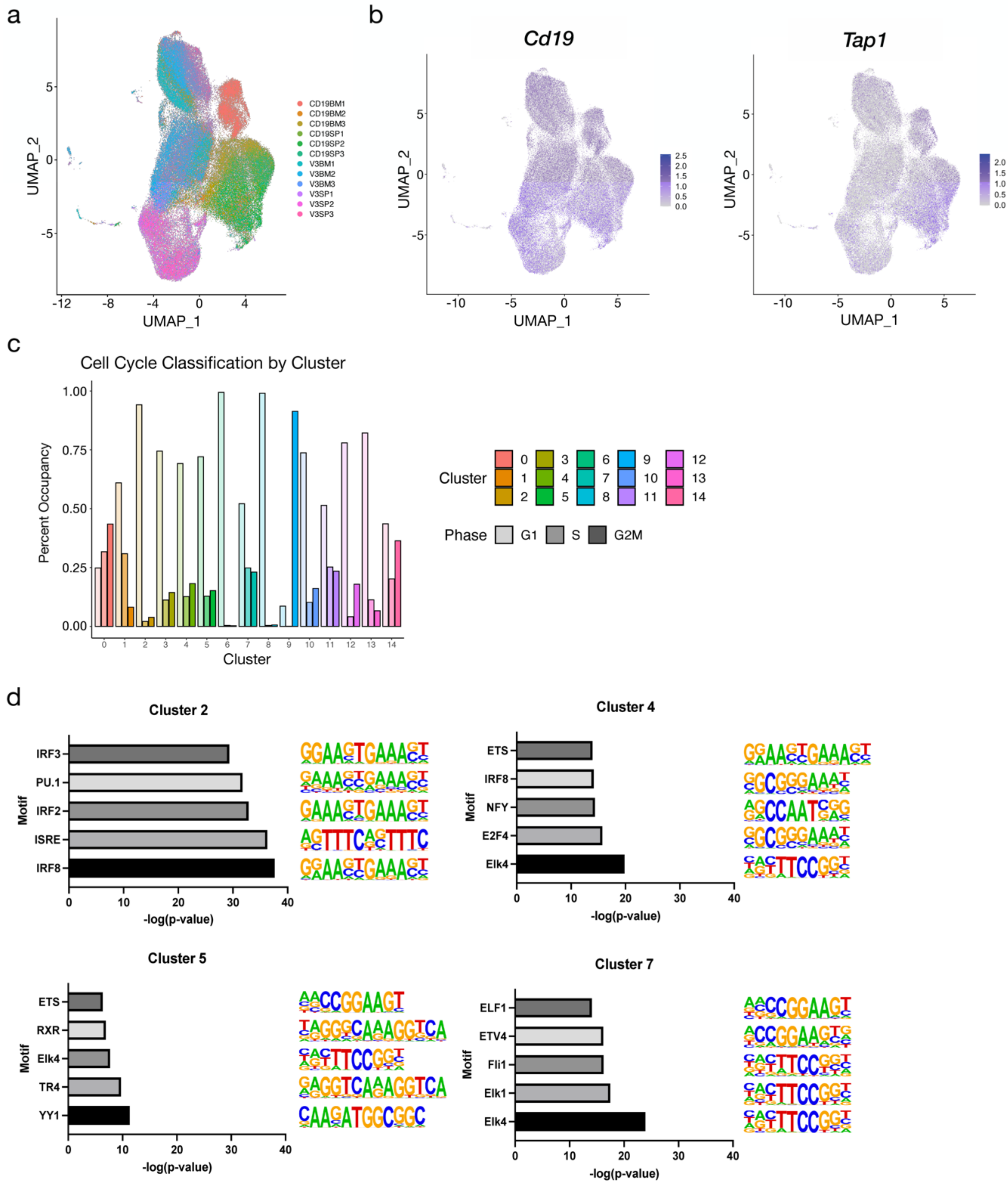

Supplementary Figure 7. Additional characterizations of clusters that emerge from single-cell expression profiling of CAR-T cell treated animals. (a) Two-dimensional UMAP plots of single cell gene expression profiles collected from mice treated with either anti-mCD19 or anti-hEGFRvIII CAR-T cell therapy, with cells color-coded by each individual mouse in the dataset. (b) UMAP plot showing expression of *Cd19* (left panel) and *Tap1* (right panel) in the single cell expression dataset. (c) Proportions of cells assigned to each cell cycle stage in each of the 15 clusters identified in the single cell expression data. (d) Transcription factor motif enrichment analysis for signature genes in clusters 2, 4, 5 and 7. Shown are -log10 transformed *p*-values for top 5 enriched motifs along with their position-weight matrix logos for each cluster.

Supplementary Figure 8

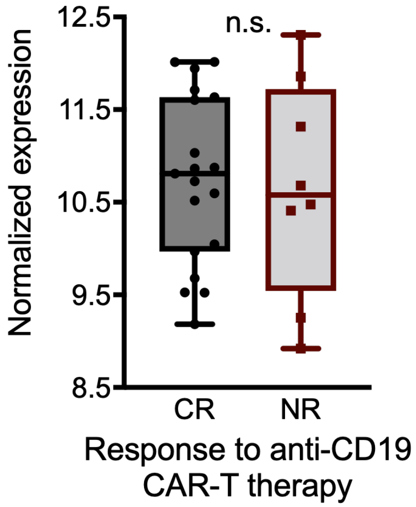

Supplementary Figure 8. Expression of HLA-E in pre-treatment samples of patients with B-ALL does not correlate with clinical outcome. Normalized expression of HLA-E in pre-treatment patient samples from complete responders (CR) and non-responders (NR) do not significantly differ. The plots are graphed from minima to maxima and all data points are overlaid. The five number summaries of the CR and NR boxplots are 9.18, 9.97, 10.8, 11.6, 12.0 and 8.92, 9.54, 10.58, 11.72, 12.31 respectively. Significance is determined using unpaired two-sided student's t-tests and P value = 0.4078.

Supplementary Figure 1a
Full Western Blots

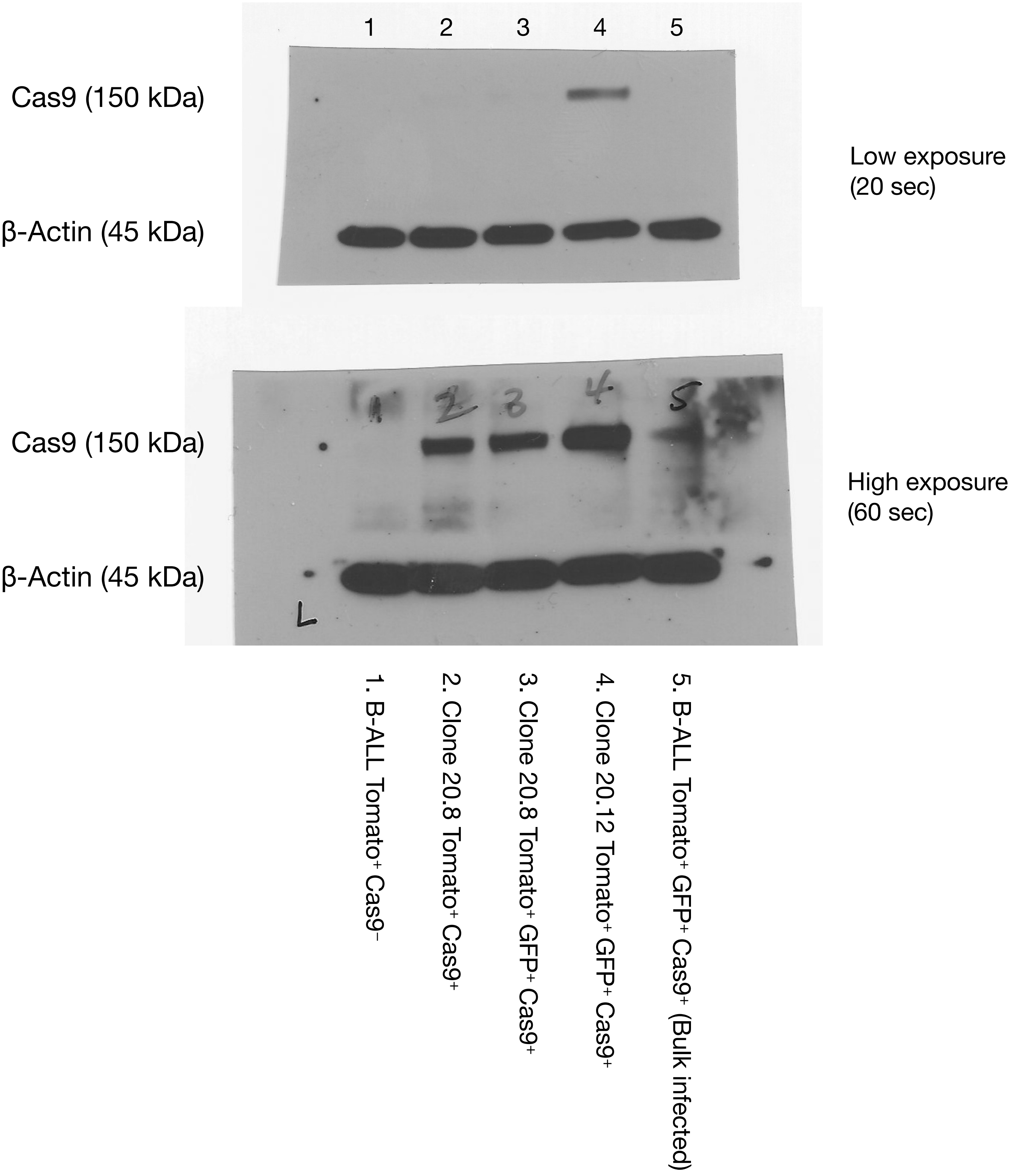

Supplementary Figure 3b
Full Western Blots

Low exposure (1 min)

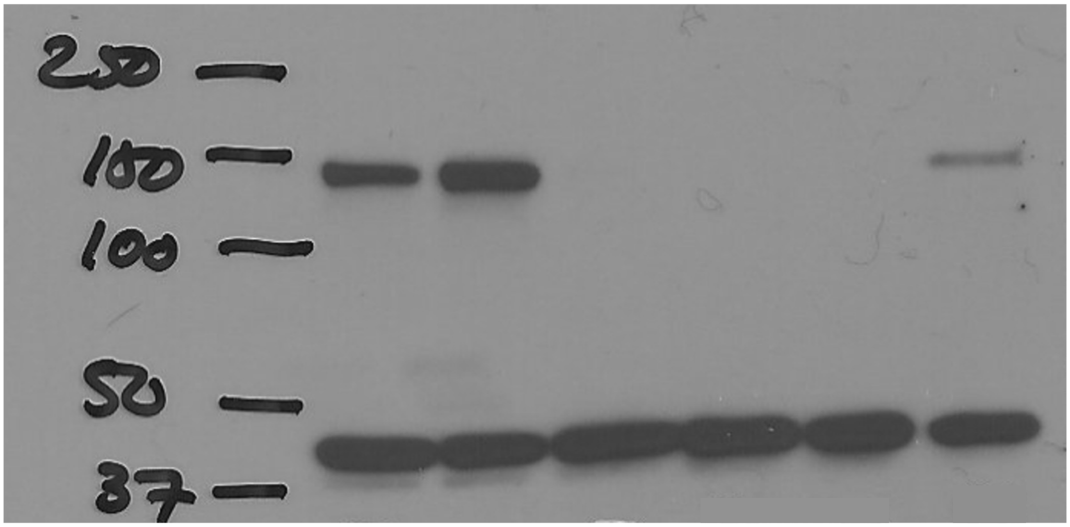

High exposure (2 min)

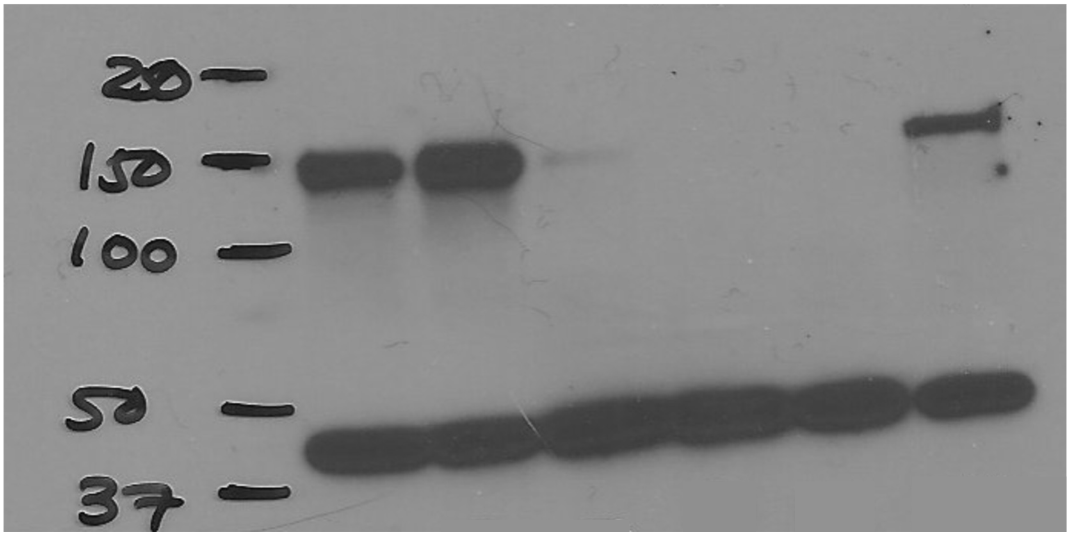

Supplementary Figure 3d
Full Western Blots

STAT1 (87 kDa)

β-Actin (45 kDa)

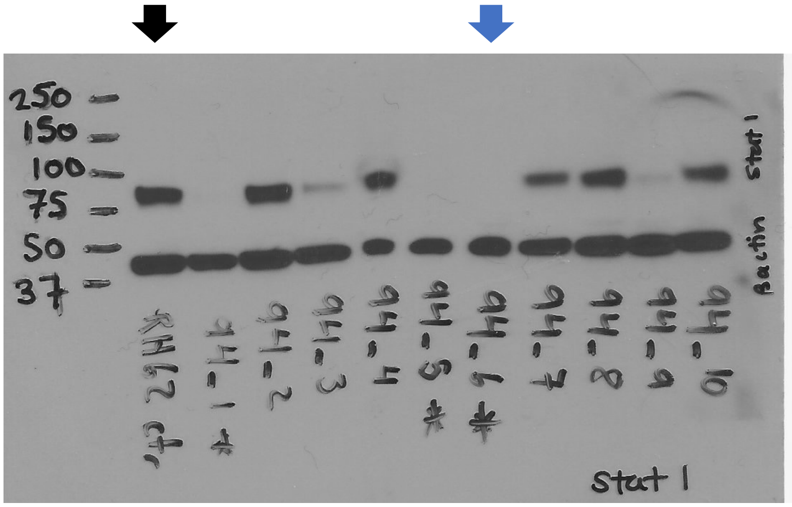

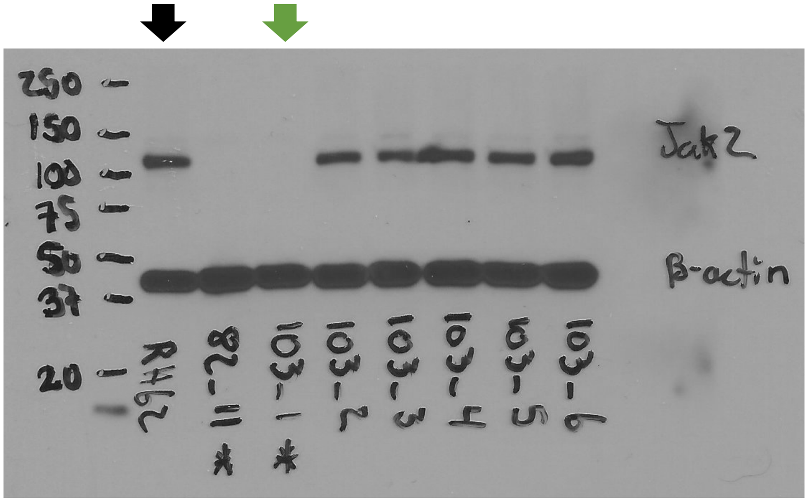

JAK2 (125 kDa)

β-Actin (45 kDa)

IFNGR1 (70-95 kDa)

β-Actin (45 kDa)

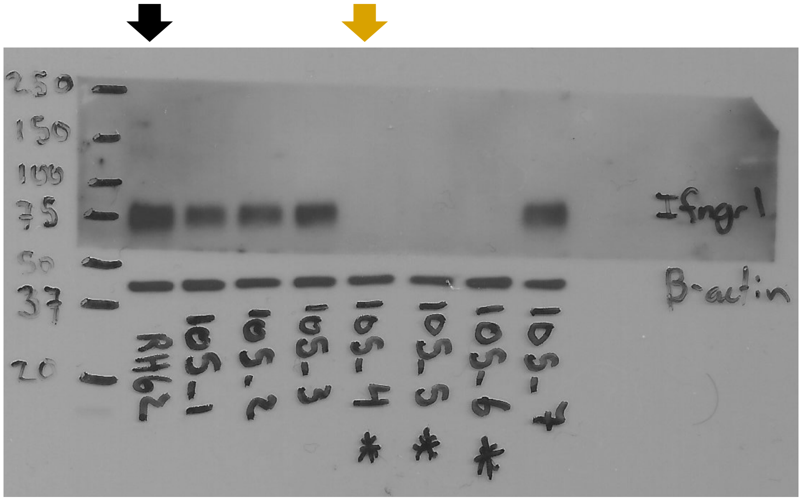

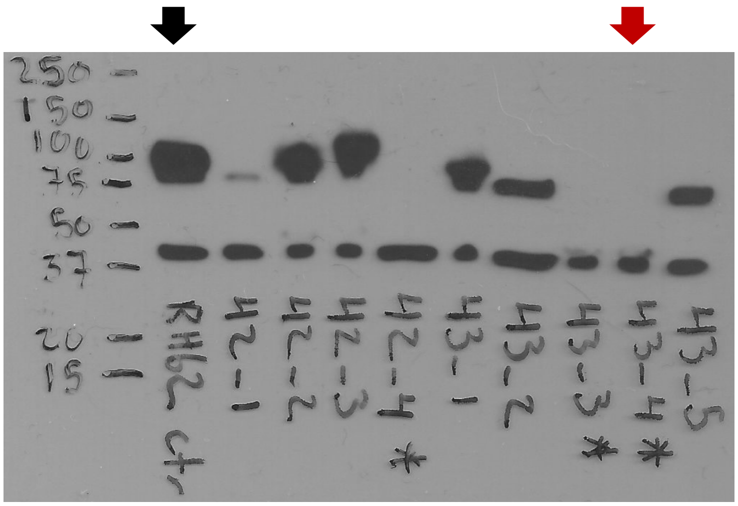

CD19 (95 kDa)

β-Actin (45 kDa)

WT CTR scc#1

Stat1$^{-/-}$ scc#2

Jak2$^{-/-}$ scc#3

Ifngr1$^{-/-}$ scc#4

Cd19$^{-/-}$ scc#5
