# Supplementary material for: Leukemia-intrinsic determinants of CAR-T response revealed by iterative in vivo genome-wide CRISPR screening
Source: Nat Commun. 2023 Dec 5;14:8048. doi: 10.1038/s41467-023-43790-2 (PMC10698189; doi:10.1038/s41467-023-43790-2)
Supplement: Supplementary file 3 — Description of Additional Supplementary Files Document [file 41467_2023_43790_MOESM3_ESM.pdf]

Description of Additional Supplementary Information file

Supplementary Data 1 – Validation screen data

Supplementary Data 2 – a) Genes comprising the 33 Sensitizer Signature described in main Figure 6a. b) Genes comprising the JAK/STAT/MHC-I 34 Signature described in 35 main figure 6b.

Supplementary Data 3 - P values for indicated figures 38 and comparisons.
