# Supplementary material for: Leukemia-intrinsic determinants of CAR-T response revealed by iterative in vivo genome-wide CRISPR screening
Source: Nat Commun. 2023 Dec 5;14:8048. doi: 10.1038/s41467-023-43790-2 (PMC10698189; doi:10.1038/s41467-023-43790-2)
Supplement: Supplementary file 7 — Reporting Summary [file 41467_2023_43790_MOESM7_ESM.pdf]

nature portfolio

Corresponding author(s): Michael T. Hemann

Last updated by author(s): 11/10/2023

Reporting Summary

Nature Portfolio wishes to improve the reproducibility of the work that we publish. This form provides structure for consistency and transparency in reporting. For further information on Nature Portfolio policies, see our Editorial Policies and the Editorial Policy Checklist.

Statistics

For all statistical analyses, confirm that the following items are present in the figure legend, table legend, main text, or Methods section.

n/a	Confirmed	
☐	☒	The exact sample size (*n*) for each experimental group/condition, given as a discrete number and unit of measurement
☐	☒	A statement on whether measurements were taken from distinct samples or whether the same sample was measured repeatedly
☐	☒	The statistical test(s) used AND whether they are one- or two-sided<br>*Only common tests should be described solely by name; describe more complex techniques in the Methods section.*
☐	☒	A description of all covariates tested
☐	☒	A description of any assumptions or corrections, such as tests of normality and adjustment for multiple comparisons
☐	☒	A full description of the statistical parameters including central tendency (e.g. means) or other basic estimates (e.g. regression coefficient) AND variation (e.g. standard deviation) or associated estimates of uncertainty (e.g. confidence intervals)
☐	☒	For null hypothesis testing, the test statistic (e.g. *F*, *t*, *r*) with confidence intervals, effect sizes, degrees of freedom and *P* value noted<br>*Give P values as exact values whenever suitable.*
☒	☐	For Bayesian analysis, information on the choice of priors and Markov chain Monte Carlo settings
☒	☐	For hierarchical and complex designs, identification of the appropriate level for tests and full reporting of outcomes
☐	☒	Estimates of effect sizes (e.g. Cohen's *d*, Pearson's *r*), indicating how they were calculated

Our web collection on statistics for biologists contains articles on many of the points above.

Software and code

Policy information about availability of computer code

Data collection	No commercial, open source, or custom code was used for this study.
Data analysis	Screen hit discovery: A series of 91 tab-delimited text files containing sample count data assigned to SKY library barcodes were produced by the Broad Institute's Genomic Perturbation Platform (GPP) using PoolQ (version 2.2.0) from three separate sequencing runs. These data were imported and organized into 12 dataframes, one for each pool of barcodes in both the invitro and in vivo experiments. This processing was done using R version 4.2.1 (R Core Team 2021), tidyverse 1.3.2. These dataframes were then used as input for differential comparison with MAGeCK test (version 0.5.9.4;) using adjust-method fdr, gene-lfc-method median and norm-method control using a list of negative control guides that target intergenic or olfactory gene sequences. Each pool included approximately 1000 negative control guides. After testing, guide and gene-level data results for each experiment and pool were imported, processed, and visualized in R using MAGeCKFlute (version 2.0.0) and tidyverse 1.3.2. For whole-genome consideration, pool data was aggregated into a single dataframe. The associations between guides and genes was done using assembled pool-level chip files. The full SKY library contains 88793 barcodes. Of these 82372 target a single gene based on our working annotation file. Barcodes that target multiple genes and genes targeted by less than 3 barcodes were deprioritized using a whitelist annotation file. Log fold change and FDR thresholds were then applied to prioritize enriched and depleted genes. All input count files, Rmd code, MAGeCK scripts and annotation files will be provided upon request.<br><br>Bulk transcriptome analysis: Paired-end RNA-seq data was used to quantify transcripts from the mm10 mouse assembly with the Ensembl version 101 annotation using Salmon version 1.3.0.70 Gene level summaries were prepared using tximport version 1.18.071 running under R version 4.0.3.45<br><br>Single cell transcriptome profiling, data processing: Sequencing data was aligned to the mm10 reference genome and converted to fastq files using bcl2fastq (v2.20.0.422). Cell count matrices were generated using cellranger (v.5.0). Matrices were analyzed by Seurat (v4.0.4) for R

(v4.0.2). Digital gene expression matrices were filtered to exclude low quality cells (< 1000 UMI, < 400 genes or > 8000 genes, > 50% mitochondrial reads). Low-quality cells were further filtered from the dataset using the variance sink method as previously described.72 Briefly, data was normalized and scaled, known cell cycle genes were regressed out.73 Principal component analysis was performed on regressed and scaled data. Standard deviation of principal components was quantified using an elbow plot, input dimensions for SNN clustering (EGFRv3 bone marrow = 35, EGFRv3 spleen = 42, CD19 bone marrow = 30, CD19 spleen = 30) at which standard deviation = 2. SNN clustering was performed to generate UMAP plots (k.param = 40, res = 0.5). Clusters containing low quality cells (50% of cells with > 10% mitochondrial reads) were removed from the dataset. After filtering, samples from bone marrow and spleen treated with EGFRv3-CAR-T or CD19-CAR-T were merged into a single dataset. Cell cycle phase was assigned using the cell cycle scoring function based on expression of known cell cycle genes.73 Merged data set was normalized and scaled, cell cycle genes were regressed out. Principal component analysis was performed, and standard deviation of principal components was quantified by elbow plot. Nearest neighbors were found (dim = 50, k.param = 40) then clustered using SNN clustering (res = 0.5). Enriched genes for each cluster were identified with the cluster marker function. Cluster occupancy was quantified for each treatment condition and phase of the cell cycle to further define therapeutic response.

Manual analysis of flow cytometric data was carried out using FlowJo software (TreeStar).

In vivo bioluminescence data was analyzed using Living Image version 4.4 software (Caliper Life Sciences).

All other statistical analyses were performed with GraphPad Prism 10 (GraphPad Software). The specific statistical tests performed are specified in figure legends. Differences are considered significant for P-values ≤ 0.05, or as indicated in the manuscript text when adjustments for multiple hypothesis testing was required.

For manuscripts utilizing custom algorithms or software that are central to the research but not yet described in published literature, software must be made available to editors and reviewers. We strongly encourage code deposition in a community repository (e.g. GitHub). See the Nature Portfolio guidelines for submitting code & software for further information.

Data

Policy information about availability of data

All manuscripts must include a data availability statement. This statement should provide the following information, where applicable:

- Accession codes, unique identifiers, or web links for publicly available datasets
- A description of any restrictions on data availability
- For clinical datasets or third party data, please ensure that the statement adheres to our policy

The RNAseq datasets generated from the BCR-ABL+ murine B-ALL model and analyzed during the current study are available in the GEO repository under the publicly available accession number GSE196143 [https://www.ncbi.nlm.nih.gov/geo/query/acc.cgi?acc=GSE196143]. The in vivo screening datasets and analysis methodology, including the input count files, Rmd code, MAGeCK scripts, MAGeCK output and various annotation files for both the primary and validation screens are available in this github repository: https://github.com/KochInstitute-Bioinformatics/Ramos_Koch_Leuk. Source data for the validation screen are provided with this paper.

Research involving human participants, their data, or biological material

Policy information about studies with human participants or human data. See also policy information about sex, gender (identity/presentation), and sexual orientation and race, ethnicity and racism.

Reporting on sex and gender	This information has not been collected for this study.
Reporting on race, ethnicity, or other socially relevant groupings	This information has not been collected for this study.
Population characteristics	This information has not been collected for this study.
Recruitment	This does not apply to our study.
Ethics oversight	This does not apply to our study.

Note that full information on the approval of the study protocol must also be provided in the manuscript.

Field-specific reporting

Please select the one below that is the best fit for your research. If you are not sure, read the appropriate sections before making your selection.

☒ Life sciences ☐ Behavioural & social sciences ☐ Ecological, evolutionary & environmental sciences

For a reference copy of the document with all sections, see nature.com/documents/nr-reporting-summary-flat.pdf

Life sciences study design

All studies must disclose on these points even when the disclosure is negative.

Sample size	For survival experiments using B-ALL cells, control mice consistently die at days 10-12 post transplant. At 5% of significance level and 80%

Sample size	power, the minimum sample size required to see at least a five day increase in survival is 2. Thus, for all survival experiments, at least 3 animals per group were included per replicate experiment. For disease suppression and validation experiments, the minimum sample size required to see at least a 50% change (absolute log2 change of 1 or more) in the target population is 3 (again, at 5% of significance level and 80% power). Thus, for all disease suppression or validation (in vivo growth competition assays), at least 3 animals per group were included per replicate experiment. For primary screens, 3-8 animals per condition were set up and at least 2 animals with adequate remaining tumor burden in the indicated organ were processed and sorted. Exclusion criteria based on read count limits for screening animals is indicated in the text. All in vitro experiments, including primary screens, were completed using a minimum of three technical replicates per group.
Data exclusions	Data included for the primary screens is shown in supplementary figure 2. Exclusion criteria based on read count limits for screening animals and in vitro conditions is indicated in the text. For validation and survival experiments, mice were excluded if the disease or CAR-T transplantation procedure was not successfully executed on days 0 or 2 of the experiment, respectively.
Replication	Aside from primary screens and RNAseq experiments, all in vitro experiments were repeated at least three times and all in vivo experiments were repeated at least twice. All attempts at replication of the included data were successful.
Randomization	Animals were transplanted with tumor cells and then randomized into experimental groups, as indicated.
Blinding	For animal studies, tumor burden, survival measurements, and tumor cell proportions were carried out by an operator who was blinded to treatment groups.

Reporting for specific materials, systems and methods

We require information from authors about some types of materials, experimental systems and methods used in many studies. Here, indicate whether each material, system or method listed is relevant to your study. If you are not sure if a list item applies to your research, read the appropriate section before selecting a response.

Materials & experimental systems

n/a	Involved in the study	
☐	☒	Antibodies
☐	☒	Eukaryotic cell lines
☒	☐	Palaeontology and archaeology
☐	☒	Animals and other organisms
☒	☐	Clinical data
☒	☐	Dual use research of concern
☒	☐	Plants

Methods

n/a	Involved in the study	
☒	☐	ChIP-seq
☐	☒	Flow cytometry
☒	☐	MRI-based neuroimaging

Antibodies

Antibodies used

Western blotting:
anti-β-ACTIN (Cell signaling, catalog# 4967S), dilution 1:1000
anti-CD19 (Abcam, catalog# ab25232), dilution 1:1000
anti-Cas9 (ActiveMotif, catalog# 61577), dilution 1:1000
anti-JAK2 (Cell Signaling Technology, catalog# 3230), dilution 1:1000
anti-IFNGR1/CD119 (R&D Systems, catalog# MAB10261), dilution 1:500
anti-STAT1 (Cell Signaling Technology, catalog# 9172), dilution 1:1000
anti-rabbit IgG HRP-linked antibody (Cell Signaling Technology, catalog# 7074), dilution 1:2000
anti-mouse IgG HRP-linked antibody (Cell Signaling Technology, catalog# 7076), dilution 1:2000
anti-rat IgG HRP-linked antibody (Cell Signaling Technology, catalog# 7077), dilution 1:2000
Rabbit anti-Armenian Hamster IgG H&L (HRP) (Abcam, catalog# ab5745), dilution 1:2000

Flow cytometry:
anti-mouse CD19-BV785 (BioLegend, catalog# 115543), dilution 1:200
anti-mouse CD8-PE/Cy7 (BioLegend, catalog# 100722), dilution 1:200
anti-mouse CD8-GFP (BioLegend, catalog# 100706), dilution 1:200
anti-human CD19-APC/Cy7 (BioLegend, catalog# 302218), dilution 1:200
anti-mouse Qa-1b-BV786 (BD Biosciences, catalog# 744390), dilution 1:200
anti-mouse Nk1.1-BV785 (BioLegend, catalog# 108749), dilution 1:200

In vivo blocking antibodies:
InVivoMAb anti-mouse IFNg antibody (Clone XMG1.2, Bio X Cell, Catalog# BE0055), not for flow cytometry or WB.
InVivoMAb anti-horseradish peroxidase control antibody (clone HRPN, Bio X Cell, Catalog# BE0088), not for flow cytometry or WB.
InVivoMAb anti-mouse NKG2A antibody (clone 20D5, Bio X Cell, Catalog# BE0321), not for flow cytometry or WB.
InVivoMAb rat IgG2a isotype control antibody against trinitrophenol (clone 2A3, Bio X Cell, Catalog# BE0089), not for flow cytometry or WB.
InVivoMAb anti-mouse NK1.1 (Clone PK136, BioXCell, catalog# BE0036), not for flow cytometry or WB.
InVivoMAb mouse IgG2a isotype control, unknown specificity (Clone C1.18.4, BioXCell, Catalog# BE0085), not for flow cytometry or WB.

Validation	Western blotting:<br>anti-β-ACTIN, tested by WB on HeLa, C2C12, C6, COS, and MvLu cells, and by recombinant Actin isoforms (manufacturer)<br>anti-CD19, tested by flow cytometry analysis of BALB/c mouse splenocytes (manufacturer)<br>anti-Cas9, tested via western blot on HEK293 cells that were transiently transfected with Myc-Tagged Cas9 or untransfected (manufacturer)<br>anti-JAK2, tested in K-562 cells that were transfected with either an siRNA against Jak2 or a control siRNA (manufacturer)<br>anti-IFNGR1/CD119, tested via western blot in our B-ALL cells while making knockout cells for this gene<br>anti-STAT1, tested via western blot on WT or STAT1 knockout A549 cells (manufacturer)<br>anti-rabbit IgG HRP-linked antibody, thoroughly validated with Cell Signaling Technologies primary antibodies (manufacturer)<br>anti-mouse IgG HRP-linked antibody, thoroughly validated with Cell Signaling Technologies primary antibodies (manufacturer)<br>anti-rat IgG HRP-linked antibody, thoroughly validated with Cell Signaling Technologies primary antibodies (manufacturer)<br>Rabbit anti-Armenian Hamster IgG H&L (HRP), tested against purified Armenian Hamster IgG (manufacturer)<br><br>Flow cytometry:<br>anti-mouse CD19-BV785, tested by flow cytometry on C57BL/6 mouse splenocytes (manufacturer)<br>anti-mouse CD8-PE/Cy7, tested by flow cytometry on C57BL/6 mouse splenocytes (manufacturer)<br>anti-mouse CD8-GFP, tested by flow cytometry on C57BL/6 mouse splenocytes (manufacturer)<br>anti-human CD19-APC/Cy7, tested by flow cytometry on human peripheral blood lymphocytes (manufacturer)<br>anti-mouse Qa-1b-BV786, tested by flow cytometry on activated splenocytes from C57BL/6 and BALB/c mice (manufacturer)<br>anti-mouse Nk1.1-BV785, tested by flow cytometry on C57BL/6 mouse splenocytes (manufacturer)<br><br>In vivo blocking antibodies:<br>InVivoMAb anti-mouse IFNg antibody, ELISA assays in in vitro co-culture experiments using B-ALL cells and recombinant IFNg<br>InVivoMAb anti-horseradish peroxidase control antibody, ELISA assays in in vitro co-culture experiments using B-ALL cells and recombinant IFNg<br>InVivoMAb anti-mouse NKG2A antibody, depletion validation by flow cytometry in blood samples from treated mice in pilot experiments<br>InVivoMAb rat IgG2a isotype control antibody against trinitrophenol, depletion validation by flow cytometry in blood samples from treated mice in pilot experiments<br>InVivoMAb anti-mouse NK1.1, depletion validation by flow cytometry in spleen samples from treated mice in pilot experiments<br>InVivoMAb mouse IgG2a isotype control, unknown specificity, depletion validation by flow cytometry in spleen samples from treated mice in pilot experiments

Eukaryotic cell lines

Policy information about cell lines and Sex and Gender in Research

Cell line source(s)	Murine cell lines: The BCR-ABL+ B-ALL cell line was generously provided by Dr. Charles Sherr. The Eμ-Myc cell line was generated in house and has been extensively published on by our group and others in previous studies (PMID: 19783987, PMID: 21029859, PMID: 24318931, PMID: 27566778). Gl261 murine glioma cells were a kind gift from David Zagzag (New York University). The KPC cell line was created and shared by A. Maitra and S. Dougan.<br>Human cell lines: 293T cells (ATCC, Catalog # CRL-3216) and Raji cells (ATCC, Catalog# CCL-86) were obtained from the American Type Culture Collection (ATCC).
Authentication	Human cell line authentication was performed by ATCC using short tandem repeat (STR) profiling. These data can be found on the manufacturer's website: 293T (ATCC, Catalog # CRL-3216); Raji (ATCC, Catalog# CCL-86)<br><br>Murine cell line authentication for both Eμ-Myc and B-ALL was previously performed in house on early frozen stocks (PMID: 21186347, PMID: 29853524, PMID: 28607179, PMID: 25737277). These cell lines were not independently authenticated again for this study.<br><br>Gl261 murine glioma cells and KPC cells were not independently authenticated.
Mycoplasma contamination	Low passage working banks of cells were sent for to IDEXX-RADIL BioAnalytics for mycoplasma testing using the IMPACT I test (Infectious Microbe PCR AmplifiCation Test). This panel tests for 23 agents: Mycoplasma spp., M. Pulmonis, Sendai virus, MHV, PVM, MVM, MPV-1,2,3, GD-7, Murine Norovirus, REO-3, EDIM, Ectromelia, LCMV, Polyoma virus, LDEV, MAD-1, 2, MCMV, K virus, MTV, and Hantaan virus. Mycoplasma testing and authentication are routinely performed. All cell lines used tested negative for mycoplasma contamination.
Commonly misidentified lines<br>(See ICLAC register)	No commonly misidentified cell lines were used in this study

Animals and other research organisms

Policy information about studies involving animals; ARRIVE guidelines recommended for reporting animal research, and Sex and Gender in Research

Laboratory animals	All mouse experiments were conducted under Institutional Animal Care and Use Committee (IUCAC)-approved animal protocols at the Massachusetts Institute of Technology (MIT). The mouse strains used in this study included either male and female C57BL/6 (The Jackson Laboratories) and either male or female NOD/SCID/IL2Rg–/– (NSG; The Jackson Laboratories) mice, as indicated for each in the mouse studies methods section of the manuscript. All experimental mice used were 8-12 weeks old. Mice were housed under

social conditions (two to five mice per cage) on a 12-hour dark/12-hour light cycle, ambient temperature 21°C±1°C, and humidity 50% ± 10%. All animals were housed in the pathogen-free animal facility of the MIT Koch Institute, in accordance with the animal care standards of the institutions. Food and water was provided ad libitum. All animal research at MIT is conducted under humane conditions with utmost regard for animal welfare. The animal care facility staff is headed by a chief veterinarian and includes a veterinary assistant, animal care technicians and administrative support. All facility staff are members of the American Association of Laboratory Animal Science. MIT adheres to institutional standards for the humane use and care of animals, which have been established to assure compliance with all applicable federal and state regulations for the purchase, transportation, housing and research use of animals.

Wild animals	Not applicable to this study. No wild animals were used in this study.
Reporting on sex	Not applicable to this study.
Field-collected samples	Not applicable to this study. No field-collected samples were used in this study.
Ethics oversight	All animal protocols undergo a strict approval process with the Institutional Animal Care and Use Committee (IACUC) at MIT. The issues of pain and distress are addressed throughout this process. We are required to perform annual literature searches for less painful alternatives to our procedures and to ensure that our research does not unnecessarily duplicate previous work. The methods of euthanasia to be used are consistent with the recommendations of the Panel on Euthanasia of the American Veterinary Medical Association.

Note that full information on the approval of the study protocol must also be provided in the manuscript.

Plants

Seed stocks	Not applicable to this study. Plants were not used.
Novel plant genotypes	Not applicable to this study. Plants were not used.
Authentication	Not applicable to this study. Plants were not used.

Flow Cytometry

Plots

Confirm that:

☒ The axis labels state the marker and fluorochrome used (e.g. CD4-FITC).

☒ The axis scales are clearly visible. Include numbers along axes only for bottom left plot of group (a 'group' is an analysis of identical markers).

☒ All plots are contour plots with outliers or pseudocolor plots.

☒ A numerical value for number of cells or percentage (with statistics) is provided.

Methodology

Sample preparation	For tumor and CAR-T cells harvested from in vivo conditions, mice are sacrificed by carbon dioxide inhalation. The spleen, bone marrow, and blood are then harvested and red blood cells are lysed by incubating in Red Blood Cell Lysing Buffer (Sigma, R7757-100mL) for 5 minutes. The resulting solution is filtered through a 40uM filter and the reaction is quenched using 45mL of phosphate buffered saline (Corning, 21-031-CV). Cells are then counted and stained, as instructed by the manufacturer. Unstained and isotype controls are also prepared in parallel. Antibodies used for flow cytomery are indicated in the text, along with their respective manufacturer and catalog number.
Instrument	Research samples were analyzed on a custom 5 laser, 16-color LSRFortessa (BD Biosciences) or on a custom 4 laser, 11 color FACS LSR II (BD Biosciences). Research samples were sorted on custom 4 laser, 11-14 color FACSAriaIII sorting machines (BD Biosciences).
Software	Data were manually analyzed using FlowJo software (TreeStar).
Cell population abundance	Cell frequencies/abundances are displayed in the accompanying figures.

Gating strategy

Figure legends specify the gating strategies used. Briefly, negative gates are set using isotype control samples or fluorophore-negative control cells, and these gates are applied to all samples in the corresponding experiment. Supplementary Figure 4d-f depict representative gating strategies for both cell sorts and analyses.

☒ Tick this box to confirm that a figure exemplifying the gating strategy is provided in the Supplementary Information.
